# T3SS effector and regulator discovery by predicting interacting partners of T3SS chaperones in *Pseudomonas aeruginosa*

**DOI:** 10.1002/pro.70551

**Published:** 2026-04-19

**Authors:** Jing Zhang

**Affiliations:** ^1^ Eugene McDermott Center for Human Growth and Development University of Texas Southwestern Medical Center Dallas Texas USA; ^2^ Department of Biophysics University of Texas Southwestern Medical Center Dallas Texas USA; ^3^ Harold C. Simmons Comprehensive Cancer Center University of Texas Southwestern Medical Center Dallas Texas USA

**Keywords:** AlphaFold, chaperon, protein‐protein interaction, structure prediction, T3SS

## Abstract

*Pseudomonas aeruginosa* is a prominent opportunistic pathogen whose virulence is closely linked to its Type III Secretion System (T3SS), a specialized apparatus that injects effector proteins into host cells. T3SS chaperones are essential for stabilizing, delivering, and regulating T3SS expression. Multiple T3SS effectors and regulators have been identified and characterized in the *P. aeruginosa* reference strains such as PAO1 and PA14. However, the full repertoire of T3SS chaperones and their interacting partners in the pan‐genomes of thousands of *P. aeruginosa* isolates remains to be explored. Here, I systematically screened over 15,000 high‐quality *P. aeruginosa* genomes to identify T3SS chaperones using structure‐assisted homology searches. Subsequently, I applied AlphaFold2 and AlphaFold3 to predict protein‐protein interactions between chaperones and other proteins in the pan‐proteome. A benchmark analysis suggests that our approach can effectively distinguish true interacting partners of T3SS chaperones on a proteome‐wide scale. Our analysis identified several high‐confidence candidate T3SS effectors and regulators, including putative lipoproteins, calcium‐binding proteins, and transcriptional regulators, that are potentially involved in host adaptation, T3SS regulation, and virulence modulation. I also found T3SS chaperone homologs that may serve as non‐cognate antitoxins or virulence regulators. These findings demonstrate the power of combining genomics with deep learning‐based structure and interaction prediction to uncover hidden components of bacterial virulence pathways and provide new targets for antimicrobial development.

## 1 INTRODUCTION

*Pseudomonas aeruginosa* is a Gram‐negative opportunistic pathogen that poses a major threat to immunocompromised individuals and patients in intensive care units (ICUs), causing a wide range of infections, including pneumonia, bloodstream infections, wound infections, and urinary tract infections (Kerr and Snelling 2009). Its pathogenicity is driven by a complex array of virulence factors that enable host colonization, immune evasion, and survival under adverse conditions (Kerr and Snelling 2009). Among these, the Type III Secretion System (T3SS) is a critical determinant of virulence, functioning as a molecular syringe that delivers effector proteins into host cells, interfering host cellular processes and immune responses (Hauser 2009). T3SS is present in many pathogenic Gram‐negative bacteria, such as *Shigella flexneri*, *Salmonella typhimurium*, and *Escherichia coli* (Cornelis 2006). The presence of an active T3SS in *P. aeruginosa* has been associated with more severe infections, increased bacterial burden, and higher mortality rates, underscoring its significance in disease progression (Moradali et al. 2017).

T3SS is a highly specialized protein secretion apparatus composed of the needle complex, the translocation apparatus, regulatory proteins, chaperones, and effector proteins (Cornelis 2006). The T3SS frequently relies on chaperones to recognize, stabilize, and properly deliver its effector proteins to host cells (Cornelis 2006; Manera et al. 2022). These small, non‐secreted proteins prevent premature aggregation or degradation of effectors within the bacterial cytoplasm, ensuring their efficient recognition and secretion through the T3SS apparatus (Cornelis 2006; Manera et al. 2022). T3SS chaperones are generally classified into three categories based on their cargo specificity and functions (Manera et al. 2022). Class I chaperones are small, acidic cytoplasmic proteins that bind directly to type III secretion substrates to prevent premature folding or aggregation and to facilitate their delivery to the secretion apparatus. Based on their association with effectors or T3SS core components, class I chaperones can be further divided into two categories, class IA and IB. Class IA chaperones are typically dedicated to their cognate cargos encoded within the same operons. Class IB chaperones interact with multiple effectors and are typically encoded adjacent to structural components of the secretion system (Manera et al. 2022). Class II and III chaperones, which are not evolutionarily related to the class I chaperones, participate in the assembly of T3SS machinery. Class II chaperones are frequently associated with the translocation apparatus, while class III chaperones, found only in some T3SS subtypes, are associated with the needle complex (Manera et al. 2022).

Beyond their canonical roles as molecular escorts, T3SS chaperones can also serve regulatory functions. For example, the *P. aeruginosa* chaperone ExsC regulates T3SS transcription by interacting with ExsD and ExsE in a calcium‐dependent signaling cascade (Horna and Ruiz 2021). Similarly, the EPEC chaperone CesT not only stabilizes multiple effectors but also interacts with the global regulator CsrA to remodel gene expression upon host cell attachment (Katsowich et al. 2017; Ye et al. 2018). Such findings reveal that T3SS chaperones are not are not passive components of the secretion process but versatile regulatory nodes that integrate environmental sensing, effector secretion, and virulence gene control.

Despite these insights, the full repertoire of T3SS chaperones and their interacting partners across the *P. aeruginosa* pan‐genome remains poorly defined. Sequence‐based approaches are limited because T3SS chaperones are short and highly divergent at the sequence level (Manera et al. 2022). However, class I chaperones share a conserved αβα sandwich fold (Manera et al. 2022), suggesting that structure‐based homology search can uncover distant homologs that sequence methods miss. Moreover, recent advances in deep learning‐based structure and interaction prediction, particularly with AlphaFold models, now allow structural inference even for uncharacterized proteins. These capabilities open new avenues for proteome‐wide discovery of T3SS‐associated proteins.

In this work, we leverage structure‐informed deep learning to systematically identify T3SS chaperone homologs and their potential interaction partners across more than 15,000 *P. aeruginosa* genomes. Our pipeline integrates structure‐assisted homology classification with large‐scale interaction screening powered by AlphaFold‐based modeling. Although studies have shown that AlphaFold‐Multimer (Evans et al. 2022) and AlphaFold3 (Abramson et al. 2024) outperform the original AlphaFold2 in modeling complex structures, our prior benchmarks (Humphreys et al. 2024; Zhang et al. 2025) indicate that these newer models tend to have less discriminative power for identifying true interactions in large‐scale screens. In contrast, AlphaFold2 yields more discriminative separation between true and false protein–protein interactions. Therefore, I used AlphaFold2 as the primary screening tool to ensure reliable discrimination of genuine chaperone–partner interactions across tens of thousands of protein pairs, and subsequently applied AlphaFold3 for high‐resolution modeling of selected complexes. This two‐tier design balances throughput, interpretability, and structural accuracy, enabling both broad discovery and mechanistic insight.

Using this strategy, we screened the *P. aeruginosa* pan‐proteome to identify T3SS chaperone homologs based on structural conservation, then predicted their interacting partners with high‐confidence interaction probabilities derived from AlphaFold2 distograms. Benchmarking against known T3SS chaperone–effector pairs suggested that AlphaFold2 accurately distinguishes true interactions from non‐specific ones, even at a low signal‐to‐noise ratio. Subsequent modeling with AlphaFold3 revealed some complexes used the conserved β‐strand‐mediated interface characteristic of known class I chaperone–effector interactions for interactions.

This integrated computational framework led to the identification of multiple high‐confidence candidate T3SS effectors and regulators, including putative lipoproteins, calcium‐binding proteins, and transcriptional regulators that may participate in host adaptation or virulence regulation. Moreover, we discovered three T3SS chaperone homologs, including DUF3156, YflI, and YbjN, that appear to have diverged functions, potentially as antitoxins or non‐canonical virulence regulators.

Together, our study establishes a scalable, interpretable, and biologically grounded approach for mapping chaperone–effector networks in bacterial pathogens. By coupling genome‐scale structural screening with deep learning–based interaction prediction, we expand the known T3SS landscape in *P. aeruginosa* and provide a generalizable computational strategy for dissecting virulence‐associated protein–protein interactions across diverse microbial species.

## 2 RESULTS AND DISCUSSIONS

### 2.1 T3SS chaperones and effectors are highly pervasive among *P. aeruginosa* strains

From over 40,000 *P. aeruginosa* genome assemblies available in the NCBI, I retained 15,536 assemblies with an N50 value exceeding 200 kb after removing duplicates in RefSeq and Genbank databases. Annotated proteins from these assemblies could be grouped into 240,283 clusters (identity >80% and coverage for both proteins >80%). 15,305 out of the 15,536 proteomes (98.5%) contained the T3SS ATPase (SctN) and at least 9 of the 10 conserved T3SS components (Hu et al. 2017), suggesting a high prevalence of T3SS in *P. aeruginosa*. Clusters were further filtered to include only proteins present in at least 50 assemblies (~0.3% of the dataset), resulting in a pan‐proteome dataset consisting of ~94 million proteins across 21,946 clusters, derived from 15,305 proteomes. Each *P. aeruginosa* genome typically encodes around 5700–6900 proteins. Among them, 5229 core proteins are shared by over 90% of genomes; in addition, most genomes encode 9%–22% auxiliary proteins it has gained through horizontal gene transfer.

**FIGURE 1:**
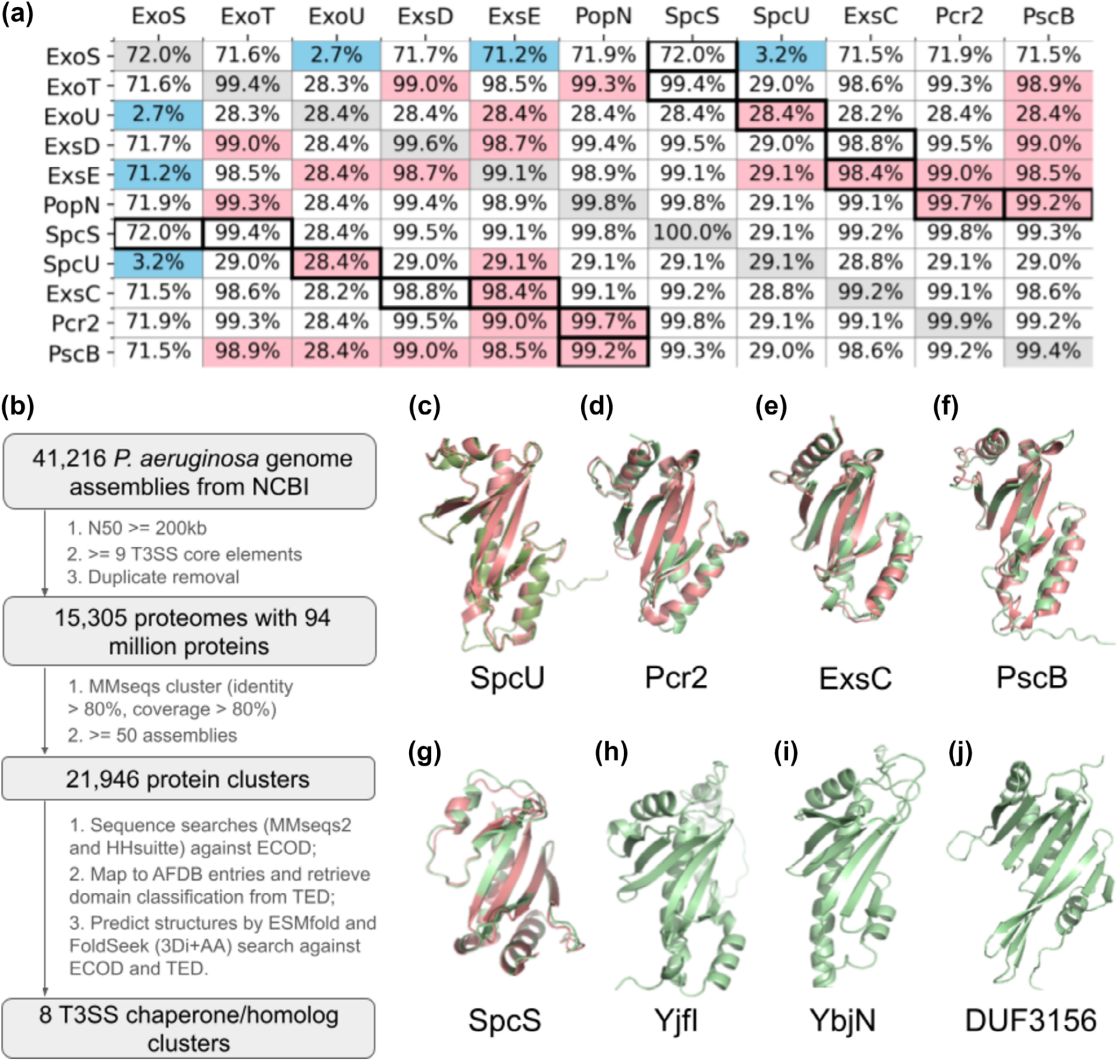
Identifying T3SS chaperone homologs from the *P. aeruginosa* pan‐genome. (a) Known chaperones and effectors are widespread in *P. aeruginosa* genomes. Each gray cell along the diagonal shows the percentage of genomes where a chaperone/effector is present; other cells show the percentage of genomes where a chaperone/effector (labeled by the X‐axis) is co‐occurring with another (labeled by the Y‐axis). Cells in black borders correspond to known chaperone–effector pairs. Cells in red indicate statistical significance (*p*‐value <0.05 after Bonferroni correction) for co‐occurring and cells in blue indicate statistical significance for avoiding each other. (b) Our workflow to identify T3SS chaperone homologs. (c–g) Predicted structures (green) and experimentally determined homologous templates (red) for known T3SS chaperones in *P. aeruginosa*. (h–j) Predicted chaperones based on structure‐assisted protein classification.

Analysis of known class I T3SS chaperones and effectors revealed their widespread distribution among *P. aeruginosa* proteomes with T3SS core components. Meanwhile, none of known effectors and chaperones exist in *P. paraeruginosa* strains that do not contain the T3SS core components (Rudra et al. 2022). Except spcU, the chaperone for exoU, all other known chaperones were detected in over 99% of the assemblies (gray cells of Figure 1a). Most of the well‐known T3SS effectors/regulators are also present in more than 99% of *P. aeruginosa* proteomes, with the exception of exoS and exoU that were identified in 72% and 28% of proteomes, respectively. Co‐occurrence of exoS and exoU was observed in approximately 400 proteomes, accounting for 2.7% of the total dataset. Fisher's exact test suggests that exoS and exoU tend not to co‐exist in the same proteome (odd ratio = 0 and *p*‐value = 0.0), consistent with previous findings suggesting mutual exclusion between these two effectors (Horna and Ruiz 2021). Meanwhile, the less toxic ExoT is also more widespread than ExoS.

While T3SS chaperones and their associated effectors are typically co‐occurring (cells with black borders in Figure 1a), a subset of proteomes contain chaperones without their cognate effectors (Table S1, Supporting Information). Notably, 108 proteomes contained the chaperone SpcU but not its cognate effector ExoU, whereas only 5 proteomes have ExoU in the absence of SpcU. These observations suggest that these chaperones may have additional effectors and/or other functional roles beyond interacting with their known effectors.

### 2.2 Identification of T3SS class I chaperones in pan‐genomes of *P. aeruginosa*

Known class I T3SS chaperones, despite their low sequence similarity, adopt a highly conserved αβα sandwich fold. I found that all structurally characterized *P. aeruginosa* chaperones, or those (Pcr2 and PscB) with close homologs (sequence identity >40%) in the Protein Data Bank (PDB), belong to the “Type III secretory system chaperone” topology group (241.1.1) in Evolutionary Classification of Protein Domains (ECOD) (Cheng et al. 2014) or the 3.30.1460.10 superfamily in the Class Architecture Topology Homology (CATH) database (Sillitoe et al. 2021) (Table S1). I obtained predicted structures for representatives of the 21,946 clusters of proteins encoded in the *P. aeruginosa* pan‐genome. I assigned the predicted structures to the ECOD and CATH hierarchies based on classifications in The Encyclopedia of Domains (TED) database (Lau et al. 2024) and additional structural similarity searches against ECOD entries (Figure 1a).

In addition to five protein clusters containing previously known chaperones (Figure 1b–f), three more clusters (Figure 1g–i) were assigned to the topology group or superfamily representing T3SS chaperones in ECOD or CATH (Sillitoe et al., 2021). Two of these, YflI and YbjN, were classified into the “Type III secretory system chaperone” topology group (241.1.1) in ECOD (Cheng et al. 2014; Schaeffer et al. 2024) and the 3.30.1460.10 superfamily in CATH. DUF3156 was classified in the ECOD “T3SS chaperone” topology group but did not map to any established superfamilies in CATH. A DALI (Holm and Sander 1993) structure comparison between DUF3156 and known T3SS chaperones yielded z‐scores above 6, supporting a significant structural similarity. Our previously developed “Domain Parser for AlphaFold Models” (DPAM) pipeline (Schaeffer et al. 2023; Zhang et al. 2023) also assigned DUF3156 to the T3SS chaperone topology group in ECOD with a probability of 0.85. While all known chaperones and their effectors are absent in *P. paraeruginosa* strains that lack T3SS, YflI, YbjN, and DUF3156‐containing protein clusters are prevalent in these T3SS‐negative strains. These findings suggest that while YflI, YbjN, and DUF3156 are homologous to class I T3SS chaperones, their functions might have diverged from recognizing T3SS effectors.

### 2.3 AlphaFold2 can identify interacting partners of T3SS chaperones

Previous studies (Humphreys et al. 2024; Pei et al. 2022; Zhang et al. 2022; Zhang et al. 2025) have shown that AlphaFold2 can be used to detect interacting partners of proteins based on concatenated multiple sequence alignments (MSAs) of protein pairs, although it was originally designed to predict protein structures (Jumper et al. 2021). Thus, I tested whether AlphaFold2 can be used to identify interacting partners of T3SS chaperones in *P. aeruginosa*. Currently, there are seven known pairs of chaperones and effectors/regulators in *P. aeruginosa*: SpcU–ExoU, SpcS–ExoS, SpcS–ExoT, ExsC–ExsE, ExsC–ExsD, PscB–PopN, and Pcr2–PopN, and they were used as positive controls for interacting partner prediction. I used 3500 pairs between the known T3SS chaperones and essential genes (Luo et al. 2021; Zhang et al. 2025) identified in *P. aeruginosa* as negative controls. These essential genes are very unlikely to be interacting partners of T3SS. The 1:500 ratio between the positive and negative control pairs allows me to approximate the true signal‐to‐noise ratio in identifying the several true partners of each chaperone among the thousands of random proteins in the *P. aeruginosa* proteome.

**FIGURE 2:**
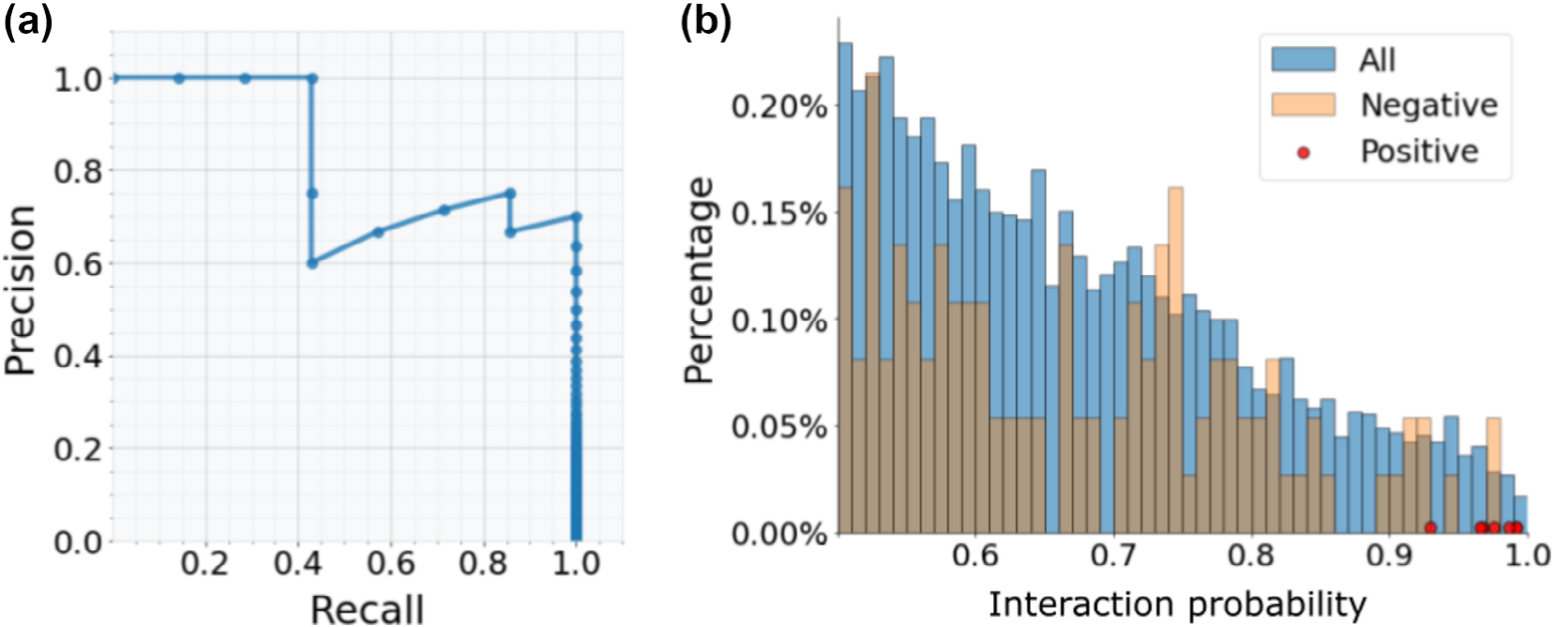
AlphaFold2 interaction probability can distinguish true PPIs involving T3SS chaperones from random pairs between T3SS chaperones and other proteins. (a) A precision–recall curve for distinguishing the positive controls from the negative controls (signal‐to‐noise ratio 1:500). Precision = TP/(FP + TP), recall = TP/P; TP is the number of positive controls above an interaction probability cutoff; FP is the number of negative controls above an interaction probability cutoff; P is the total number of positive controls. (b) Distribution of interaction probabilities among positive controls (red dots), negative controls (orange bars) and all protein pairs (blue bars) I screened.

As in our previous studies, I used interaction probabilities derived from predicted distograms of AlphaFold2 as an indicator of protein–protein interaction (PPI) (Humphreys et al. 2021; Humphreys et al. 2024), and I tested whether such predicted interaction probabilities can distinguish the positive controls from the negative ones and monitored the performance using a precision versus recall curve (Figure 2a). The method can achieve a recall of 40% and a precision of 100% at an interaction probability cutoff of 0.98. The lowest interaction probability for positive controls is 0.93, while only 3 of the 3500 negative control pairs got predicted interaction probability above 0.93, resulting in a precision of 70% (7/(7 + 3)) at a recall of 100%. It is worth noting that AlphaFold2 was trained only on monomeric proteins and thus its ability to recognize true interacting partners of T3SS chaperones is not a result of memorizing the training data; instead, AlphaFold2 captures meaningful coevolutionary and physicochemical signals and provides effective discrimination between true PPIs and non‐interacting pairs, even at a low signal‐to‐noise ratio (Humphreys et al. 2021; Humphreys et al. 2024). It is worth noting that the signal‐to‐noise ratio for predicting true interacting partners of T3SS chaperones might approach 1:5000 (*P. aeruginosa* genome encodes over 5000 proteins), and the expected precision could be much lower (about 20% at a recall of 100% for the positive controls).

Encouraged by the success in the above benchmark study, I expanded the screening to all other proteins. For each pair of chaperon/non‐chaperon protein clusters, I selected proteins within the same proteome and prioritized reference genomes or assemblies with higher N50. After excluding pairs with cumulative sequence length >2500 aa (computationally prohibitive) and pairs with very shallow paired MSAs lacking coevolutionary signal, I retained 157,376 pairs for screening. 7778 (~5%) of these pairs displayed interaction probabilities above 0.5. Figure 2b showed the distribution of interaction probabilities (≥0.5) for negative controls, positive controls, and all pairs included in the screening. Only two negative controls (0.06%) have obtained high interaction probability (≥0.95) while all positive controls except the ExsC–ExsE pair have interaction probabilities above 0.95. Based on these observations, I considered interaction probability above 0.95 as the cutoff to select candidate PPIs between T3SS chaperones and potential T3SS effectors/regulators. A total of 180 candidates were selected, including 132 candidates involving known T3SS chaperones.

### 2.4 A conserved interaction mode between T3SS chaperones and effectors

**FIGURE 3:**
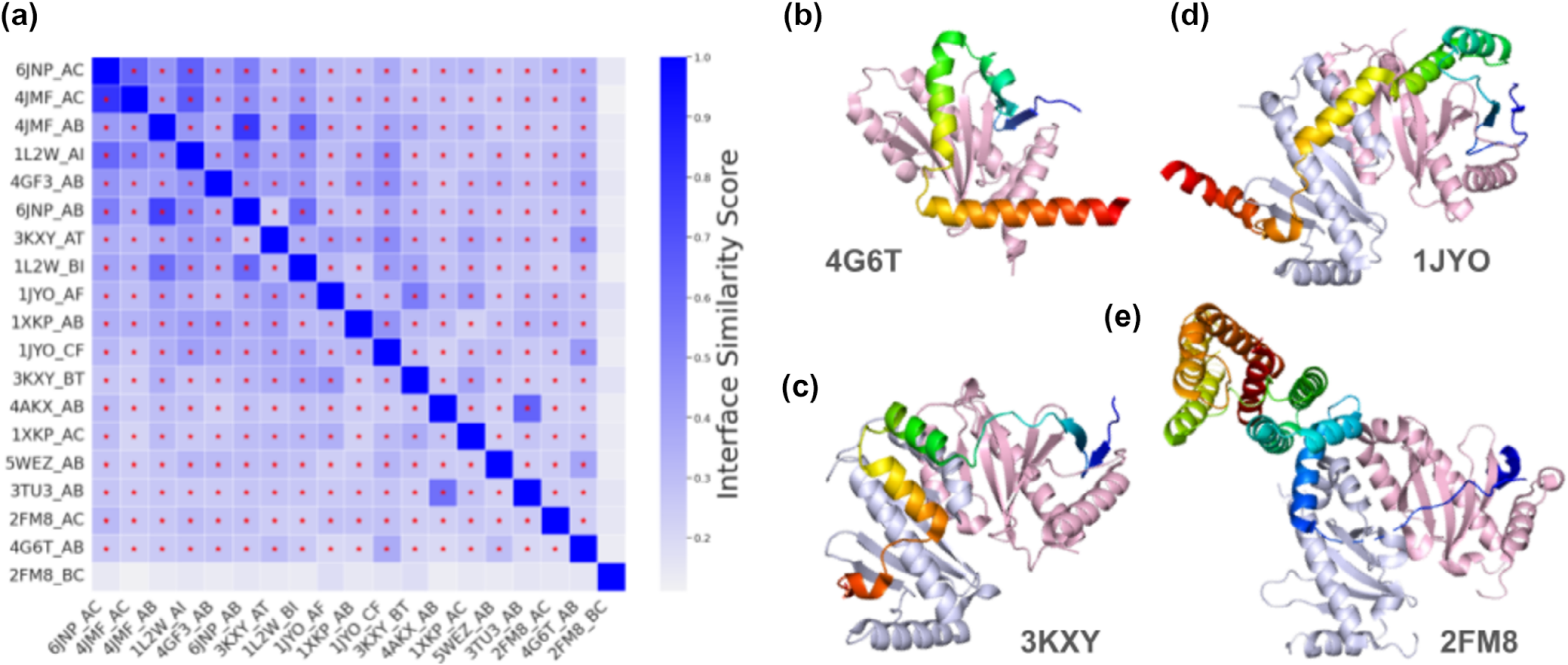
T3SS chaperone–effector complexes adopt similar interaction modes. (a) Interface similarity among experimentally determined chaperone–effector complex structures, quantified using iAlign. Structure 1ttw was excluded because the default iAlign settings do not detect peptides shorter than 25 amino acids. Darker color indicates higher similarity and the red stars mark pairs of chaperone–effector complexes showing statistically significant similarity in their interfaces. (b–e) Representative structures of chaperone–effector complexes. The chaperones are colored in light pink and blue white. Each effector is colored in a rainbow from the N‐terminus to the C‐terminus. The PDB id is labeled below each complex.

To further refine the candidate partners of T3SS chaperones, I modeled their 3D structures by AlphaFold3 and compared them against experimental structures of T3SS chaperone–effector pairs. Examination of 13 experimental structures involving class I chaperones and their cognate effectors/regulators revealed a high degree of structural similarity at the interaction interfaces. All but one pair (2FM8 (Lilic et al. 2006): InvB–SipA) showed significant interface similarities (Gao and Skolnick 2010) (Figure 3a). In most cases, the interaction between a chaperone and an effector is mediated by β‐strand pairing between the N‐terminal β‐strand of the chaperone and a β‐strand near the N‐terminal of the effector, followed by interactions between hydrophobic residues on the chaperone's β‐sheet and the unfolded extended N‐terminal region of effectors (Figure 3b) (Janjusevic et al. 2013).

In addition to functioning individually, chaperones can form dimers to bind larger effectors. In such case, the N‐terminal β‐strands of both chaperones participate in the chaperone–effector interface and the effector is unfolded, wrapping around the chaperone dimer and binding the two N‐terminal β‐strands of the chaperones on the opposite side of the dimer (Figure 3c (Vogelaar et al. 2010) and Figure 3d (Stebbins and Galan 2001)). This similar binding mode involving the N‐terminal β‐strand and hydrophobic residues on the β‐sheet of the chaperone is used by all the complexes of chaperone dimers and effectors. In the aforementioned InvB–SipA complex showing lower similarity to the rest (Figure 3e), one of the chaperone InvB interacts with the effector SipA through the N‐terminal β‐strand and hydrophobic interactions with the unfolded N‐terminus of the effector, while the other chaperone interacts with a folded C‐terminal domain of the effector, engaging more polar contacts in adjacent helices. Previous studies have suggested that such interactions with the folded domain of SipA contribute to the overall stability of the InvB–SipA complex (Lilic et al. 2006). However, the conventional chaperone–effector interface between SipA and one copy of the chaperones remains essential for strong binding and secretion competency (Lilic et al. 2006). Overall, β‐strand‐mediated interactions between chaperones and effectors appear to represent a structural signature of class I chaperone–effector recognition.

Therefore, I considered the involvement of the N‐terminal β‐strands of the chaperones as a criterion to prioritize the candidate chaperone–effector/regulator complexes. I used AlphaFold3 to model the 180 candidates passing the AlphaFold2 interaction probability cutoff. It is worth noting that AlphaFold3 is optimized to model complex structures for protein pairs that are known to interact, even with shallow input MSAs (Abramson et al. 2024). While not more suitable than AlphaFold2 in distinguishing true PPIs from random pairs (Zhang et al. 2025), it is suitable for modeling the complex structures between T3SS chaperones and candidate effectors and evaluating whether their PPI interfaces resemble known chaperone–effector pairs.

**FIGURE 4:**
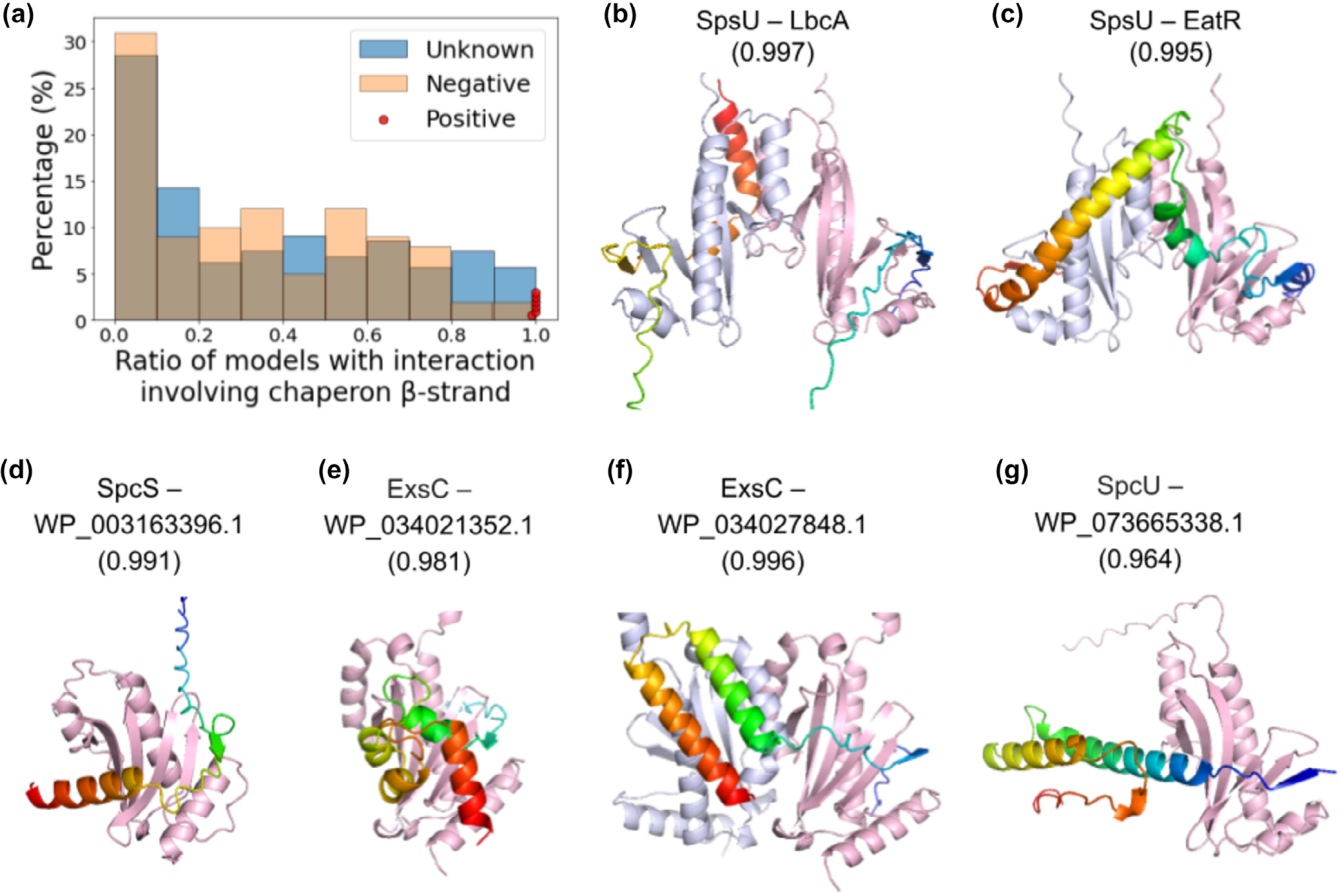
Confidently predicted PPIs between known T3SS chaperones and putative T3SS regulators or effectors. (a) Distribution of the fraction of AlphaFold3 models where T3SS chaperones use their N‐terminal β‐strands to interact with other proteins. The involvement of these N‐terminal β‐strands in the interactions can be used as a criterion to select confident predictions. (b–g) Confident predictions modeled by Alphafold 3 involving T3SS chaperones. The chaperones are colored in light pink or blue white. The putative effectors or regulators are colored in a rainbow from the N‐termini to C‐termini. The gene names or accessions of the chaperones and the effectors/regulators are labeled above their complex structures. The numbers in the parenthesis are the Alphafold2 interaction probability.

Different interaction modes between chaperones and effectors/regulators have been observed in available PDB structures. In some cases, effectors or regulators associate with two chaperones (e.g., PDB: 1jyo), whereas in others they interact with a single chaperone (e.g., PDB: 3tu3). Chaperones typically bind to the N‐terminal region of effectors, while information on their interactions with other regulatory proteins remains limited. Therefore, to capture possible interaction patterns, I modeled the potential chaperone–effector/regulator complexes under several conditions: with two chaperones or one chaperone; with the whole effector protein or only the N‐terminal segment of an effector. I also used multiple random seeds and generated 5 models for each random seed, resulting in hundreds of models for each candidate chaperone–effector/regulator complex. Similarly, I modeled the complexes between the T3SS chaperones and the positive and negative control pairs. I found that all the complexes between positive controls and T3SS chaperones almost always involve the N‐terminal β‐strands of the chaperones (red dots in Figure 4a). In contrast, predicted complex structures between T3SS chaperones and 100 randomly sampled negative controls rarely involve these β‐strands at the PPI interfaces (orange bars in Figure 4a): only 4 negative controls are predicted to interact with the β‐strands of the chaperones at high frequency (>80% of all models).

Using both the AlphaFold2 interaction probabilities and the AlphaFold3 complex structures, I selected confidently predicted interactions of T3SS chaperones and T3SS effectors/regulators passing either of the following two empirical criteria: (1) AlphaFold2 interaction probability above 0.98, which represents a stringent cutoff without relying on prior structural information; (2) AlphaFold2 interaction probability above 0.95 and over 80% of the AlphaFold3 complex structures involve the chaperone's N‐terminal β‐strand, a region known to mediate binding in T3SS chaperone–effector interactions. As a result, 44 confident predictions were selected (Table S2), in addition to positive controls. Meanwhile, I also identified 11 high contact probability interactions involving T3SS homologs (YjfI, YbjN, and DUF3156, the homologs shown in Figure 1h–j adopting the same fold as T3SS chaperones). The confidently predicted interacting partners of T3SS chaperones might represent novel T3SS effectors or T3SS regulators. Below I discussed six confident predictions and highlighted their potential biological implications. Future experimental validation of these predictions could lead to new insights in *P. aeruginosa* pathogenicity and pave roads for the treatment of *P. aeruginosa* infection.

### 2.5 Predicted novel T3SS effectors or regulators

Some predicted interactors of T3SS chaperones are possibly novel regulators of T3SS. One example is LbcA (WP_003135130.1), which is predicted to interact with SpcU by AlphaFold2 with interaction probability of 0.997 (Figure 4b). In 44% of the AlphaFold3 models of the LbcA–SpcU complex, their interaction is mediated by the N‐terminal β‐strand of the chaperone SpcU. Previous studies suggested that LbcA may serve as a scaffold for CtpA and its substrates to promote the CtpA‐dependent proteolysis. CtpA belongs to the carboxyl‐terminal processing proteases (CTP), which are widely conserved and broadly involved in protein quality control, stress response, and virulence regulation in several bacteria (Sommerfield and Darwin 2022). In *P. aeruginosa*, CtpA is shown to be essential for T3SS function (Seo and Darwin 2013), but the molecular mechanism for such functional association remains unclear. Our prediction connecting SpcU with LbcA raises the possibility that LbcA may help to recruit CtpA to the vicinity of T3SS assembly sites, thereby linking CtpA to T3SS function and explains its involvement in bacterial virulence.

In addition, SpcU is predicted to interact with a sigma‐54‐dependent transcriptional regulator EatR (WP_011666549.1) (Figure 4c), showing a high AlphaFold2 interaction probability (0.995) and a typical PPI interface involving the N‐terminal β‐strand of SpcU. EatR has been shown to regulate genes involved in ethanolamine metabolism, which is important when *P. aeruginosa* uses ethanolamine as a carbon and nitrogen source (Lundgren et al. 2016). Ethanolamine is abundant in host tissues, and its utilization can support persistence and growth in host environments. It is not uncommon for T3SS chaperones to be linked to transcription regulators, especially those regulating the expression of genes associated with host infection (Katsowich et al. 2017; Urbanowski et al. 2005). The predicted interaction between SpcU and EatR might contribute to the regulation of gene expression upon host infection, allowing *P. aeruginosa* to adapt to the host environment and utilize host nutrients.

Interestingly, I predicted interactions between T3SS chaperones and four short proteins that might represent novel T3SS effectors or regulators (Figure 4d–g). All of them are present only in a small fraction of *P. aeruginosa* genomes encoding the core T3SS components. None of the T3SS‐negative *P. paraeruginosa* genomes encode these proteins. For example, a peptide of 51 residues (WP_003163396.1) (Figure 4d), annotated as “dihydropyrimidinase,” is predicted to interact with SpcS (AlphaFold2 interaction probability 0.991) through its N‐terminal β‐strand. Typical dihydropyrimidinases contain 400–500 residues, and this fragment, possibly originated from a dihydropyrimidinase, might have adapted a new function as a T3SS effector. Interestingly, one short protein (WP_034021352.1) predicted to interact with ExsC (AlphaFold2 interaction probability 0.98) contains an EF‐hand domain (Figure 4e). EF‐hand domains typically bind calcium ions and are predominantly found in Eukaryotes where calcium is used as a universal second messenger (Kretsinger 2013). Possibly originated from Eukaryotes, this EF‐hand domain containing protein might be delivered to host cells through T3SS and interfere with host calcium signaling. Alternatively, this protein might serve as a regulator for bacterial secretion activity by sensing the change of calcium concentration upon host infection. Interestingly, ExsC's function is known to be controlled by calcium concentration, and this EF‐hand domain containing protein might contribute to the regulation of ExsC function.

### 2.6 High‐confidence interactions involving T3SS chaperone homologs

**FIGURE 5:**
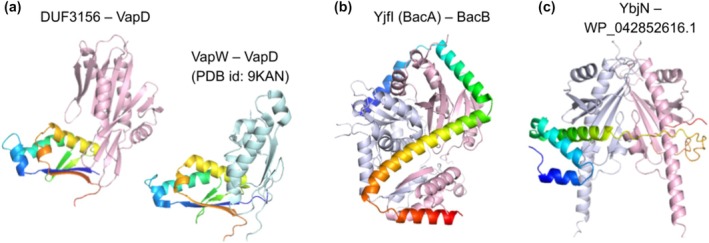
Confidently predicted PPIs between T3SS chaperone homologs and other proteins. The chaperone homologs are colored in light pink or blue white. The binding partners are colored in a rainbow from the N‐termini to C‐termini. The gene names or accessions of the chaperones and the effectors/regulators are labeled above their complex structures.

The T3SS chaperone homologs I identified might not function as T3SS chaperones, and confidently predicted interactions between them and other proteins could shed light on their functions. I predicted a confident interaction (AlphaFold2 interaction probability 0.997) between DUF3156, a T3SS chaperone homolog, and VapD (Figure 5a, left), an identified toxin and virulence factor in *Haemophilus influenzae* (Ren et al. 2012). This VapD in *P. aeruginosa* shares a high sequence identity (~42%) with *Haemophilus influenzae*, which was shown to promote intracellular survival (Gilep et al. 2025; Ren et al. 2012). VapD only exists in a few *P. aeruginosa* genomes (59 out of 15,305), suggesting the possible horizontal transfer of VapD into *P. aeruginosa* from other sources. The toxin VapD is known to be associated with antitoxins such as VapW, VapY, and VapX (Gilep et al. 2025), but these known antitoxins are not identified in many genomes harboring VapD (Gilep et al. 2025), including the *P. aeruginosa* proteomes containing VapD. Interestingly, the predicted PPI interface between DUF3156 and VapD overlaps with the experimentally characterized interface between VapW and VapD (Figure 5a, right) (Gilep et al. 2025). The identification of DUF3156 as potential VapD‐interacting partners suggests that DUF3156 may function as antitoxin of VapD. It might neutralize the toxicity of VapD inside the bacteria and possibly could escort VapD to periplasm, extracellular space, or host cells. This finding is intriguing: it not only suggests a possible function of DUF3156, but also provides new insights into the pathogenicity of VapD.

I also identified the interaction (AlphaFold2 interaction probability 0.981) between BacA (PA3732 from the same protein cluster as YjfI) and BacB (PA3731) from the same operon (Figure 5b). Previous studies have associated BacB with biofilm formation in *P. aeruginosa* (Mace et al. 2008; Vilain et al. 2004). Knockout of both BacA and BacB is shown to result in a decrease in rhamnolipid (biosurfactants produced by *P. aeruginosa*) secretion, swarming motility and virulence (Wallart et al. 2024). *ΔbacA* also results in a significant increase in the abundance of proteins in the same operon including BacB (Wallart et al. 2024). The predicted interaction between BacA and BacB is consistent with this strong functional association between them, but the molecular mechanism for their involvement in biofilm formation remains to be investigated.

Finally, I predict an interaction (AlphaFold2 interaction probability 0.995) between YbjN and a hypothetical protein (WP_042852616.1) (Figure 5c). YbjN is known to play important roles in regulating motility, biofilm formation, and survival under stress conditions in *E. coli* (Chen et al. 2006; Wang et al. 2011). The predicted interaction between YbjN and this hypothetical protein suggests that the latter may also be involved in the above process. Interestingly, while the most confident PPIs by AlphaFold2 interaction probability involving known T3SS chaperones are frequently mediated by the N‐terminal β‐strand (Figure 4), resembling the known chaperone–effector PPI interfaces, the most confident PPIs involving the T3SS chaperone homologs frequently adopt different interaction modes (Figure 5). This difference suggests that these T3SS chaperone homologs have diverged to carry out different, but possibly related functions.

## 3 CONCLUSIONS AND DISCUSSIONS

AlphaFold has emerged as a powerful tool in modeling the 3D structures of protein complexes and detecting interacting partners of proteins de novo. Using *P. aeruginosa* as an example, I demonstrated that such methods can effectively detect the several true interacting partners of T3SS chaperones among the thousands of proteins encoded in the bacterial genome. Using a computational pipeline that combines the identification of T3SS chaperone homologs based on sequence and structure similarities with the detection of interacting partners by AlphaFold, I identified putative T3SS effectors, T3SS regulators, and other virulence‐related PPIs in the *P. aeruginosa* pan‐genome. I expect my results to provide valuable insights into the pathogenicity of *P. aeruginosa* and shed light on the development of treatment strategies against this pathogen. Application of a similar pipeline to other human pathogens could provide even more insights into the T3SS‐mediated bacterial virulence, but the efficiency of this pipeline in other organisms remains to be explored.

Meanwhile, although AlphaFold provides valuable static models of chaperone–effector complexes, these predictions cannot capture the dynamic and context‐dependent nature of their interactions within the cellular environment. To elucidate the conformational and dynamic features of chaperone–effector complexes, complementary approaches such as molecular dynamics (MD) simulations are useful for revealing conformational flexibility, binding stability, and transient contact networks that underlie recognition. Integrating AlphaFold predictions with MD simulations and experimental data will further advance our understanding of T3SS chaperone–effector interactions and may guide the development of therapeutic strategies to prevent severe pathogenic infections.

## 4 MATERIALS AND METHODS

### 4.1 Prepare protein dataset for screening

*Pseudomonas aeruginosa* assembly metadata was obtained from NCBI using the command “datasets summary genomes ‐taxon ‘*Pseudomonas aeruginosa*’ ‐‐annotated ‐‐exclude‐atypical.” From 41,216 initial assemblies, I removed redundant sequences of the same *P. aeruginosa* isolate and selected 15,536 high‐quality genomes with N50 values >200 kb. I prioritized gnomes in RefSeq over GenBank and retained only the most recent versions. I downloaded annotated proteins for these genome assemblies and clustered them using MMseqs2 (Steinegger and Söding 2017) with command “mmseqs easy‐cluster all_proteomes.fasta all_proteomes_c80i80 /scratch ‐‐threads 64 ‐‐min‐seq‐id 0.8 ‐c 0.8.” The command used default coverage mode, requiring cluster representatives and their associated members to have ≥80% sequence identity and ≥80% coverage, which yielded approximately 240,283 clusters.

To verify the presence of T3SS in each genome, I searched the 10 ultra‐conserved T3SS components previously identified (Hu et al. 2017) against the cluster representative proteins by MMseqs2 and counted the number of T3SS components in each genome assembly. I retained 15,305 out of the 15,536 proteomes (~98.5%), ensuring that each contained T3SS ATPase (SctN) and at least 9 of the 10 conserved T3SS components. Clusters were further filtered to include only proteins present in no fewer than 50 assemblies (~0.3% of the dataset). The final pan‐proteome dataset comprises around 94 million proteins across 22,248 clusters, derived from 15,305 proteomes. The associations between components shown in Figure 1a were computed by fisher_exact test in the scipy.stats package.

### 4.2 Identify T3SS class I chaperone homologs

Despite low sequence similarity, class I T3SS chaperones share a conserved fold. I found that currently known class I T3SS chaperones in *P. aeruginosa* (SpcU, SpcS, ExsC, Pcr2, and PscB) all belong to the “Type III secretory system chaperone” topology group (241.1.1) in the ECOD database (Schaeffer et al. 2024) and the “3.30.1460.10” superfamily in the CATH (Sillitoe et al. 2021) database. Based on this observation, I selected cluster representatives with 60–250 residues and used multiple strategies to find if any can be assigned to the ECOD “241.1.1” topology group or CATH “3.30.1460.10” superfamily.

First, I searched their sequences against the ECOD database by MMseqs2 (command: mmseqs easy‐search [query_file] [db_file] [output_file] /tmp ‐‐cov‐mode 0 ‐c 0.8), retaining only hits showing more than 80% coverage for the query and e‐value below 0.0001; for queries without qualified MMseqs2 hits, I used HHsuite with command “hhblits ‐cpu 8 ‐i [fasta_input_fn] ‐oa3m [fasta_input_fn].a3m ‐d UniRef30_2022_02 && addss.pl [fasta_input_fn].a3m [fasta_input_fn].a3m.ss ‐a3m && mv [fasta_input_fn].a3m.ss [fasta_input_fn].a3m && hhmake ‐i [fasta_input_fn].a3m ‐o [fasta_input_fn].a3m.hhm && hhsearch ‐i [fasta_input_fn].a3m.hhm ‐d ecod_v291 ‐o [fasta_input_fn].a3m.hhsearch ‐cpu 8” to search against ECOD70 (representative domains filtered at 70% sequence identity) entries, retaining hits showing HHsearch (Steinegger et al. 2019) probabilities above 80%. Second, I searched their sequences against the AlphaFold protein structure DataBase (AFDB) (Varadi et al. 2022) using MMseqs2, retaining only hits showing more than 80% coverage for the query and e‐value below 0.0001 (command: mmseqs easy‐search [query_file] [db_file] [output_file] /tmp ‐‐cov‐mode 0 ‐c 0.8); I then selected the top hit for each query and retrieved domain classification information for these hits from the TED database (Lau et al. 2024).

Finally, for proteins without homologs detectable by MMseqs2 in AFDB, I used their sequences to search against the TED50 database using FoldSeek (3Di + AA mode, e‐value <0.0001, and hit coverage >70%) (Van Kempen et al. 2024) with the ProstT5 protein language model (Heinzinger et al. 2024) that predict 3D structure tokens from protein sequences. I also used ESMFold (Lin et al. 2023) to predict their structures (command: CUDA_VISIBLE_DEVICES = 0 python esmfold_inference.py ‐i [input_fastafile] ‐o [output_dir] ‐m [model_dir]) and applied FoldSeek (3Di + AA mode, e‐value <0.0001, and hit coverage >70%) to search these predicted structures against the 3D structures in TED50 and ECOD databases, respectively. In any of the above approaches, I kept the top hit and examined if it belonged to the CATH “3.30.1460.10” superfamily or ECOD “241.1.1” topology group.

### 4.3 Prepare inputs for PPI screening

Among 22,248 protein clusters present in more than 50 genomes, 8 clusters are homologous to T3SS chaperones. To detect possible interacting partners of them, I paired each T3SS chaperone cluster with every non‐chaperone protein cluster. I selected a pair of proteins from each pair of protein clusters (one per cluster), ensuring the pair is encoded by the same genome. If there were multiple protein pairs from a cluster pair, I prioritized the pair from the genome of a popular *P. aeruginosa* strain based on Pseudomonas genome DB (Winsor et al. 2016) or a genome with higher N50 which indicates continuity of genomic assemblies.

To build the paired MSA for each protein pair, I utilized all the *P. aeruginosa* proteomes and representative proteomes encoded by genomes of other Prokaryotes. I selected genomes using information at https://ftp.ncbi.nlm.nih.gov/genomes/GENOME_REPORTS/prokaryotes.txt (Oct. 2023), choosing up to 5 assemblies per species based on their N50 values. I detected homologs for each query protein in each proteome by MMseqs2, and I selected the closest hit for each query protein, requiring an e‐value of less than 0.0001 and coverage above 50%. Based on the pairwise sequence alignment between the query and the best hit in each proteome, I built the MSA for each protein. Based on the MSAs for a pair of proteins, I generated paired MSAs by concatenating protein sequences encoded by the same genomes. I removed redundancy from the paired MSAs by filtering at 95% sequence identity by HHfilter (hhfilter ‐i95) (Steinegger et al. 2019). Due to the limitation in GPU memory, I excluded protein pairs with cumulative size above 2500 residues. I also removed protein pairs with paired MSA depth below 2 after filtering by 95% sequence identity due to limited co‐evolutionary signals the alignments can afford. As a result, 157,376 pairs were included in our screen.

### 4.4 Screen the interacting partners for T3SS chaperone homologs

To predict interactions between T3SS chaperones and their potential binding partners, I used AlphaFold2 (Jumper et al. 2021) distributed with the ColabFold v1.5.5 (Mirdita et al. 2022) package with the options ‐‐model‐type alphafold‐ptm and ‐‐model_num 3, along with our custom MSAs. As detailed in our previous work (Humphreys et al. 2021; Humphreys et al. 2024; Zhang et al. 2025), residue–residue contact probability was calculated as the sum of the probabilities for the distance bins under 8 Å for each residue pair. And for any given pair of proteins, the matrix of contact probability of residue pairs is of the shape L1 + L2 by L1 + L2, where L1 and L2 are the length of two proteins, respectively. To investigate inter‐protein contacts, I extracted the submatrix [:L1][L1: L1 + L2] and the highest values were used as the protein interaction probability. The residue–residue contact probability has been also introduced in AlphaFold 3 output. Compared with pTM, which emphasizes global structural accuracy, and ipTM, which assesses the accuracy of the predicted relative positioning of subunits in a protein–protein complex using full‐length proteins, the interaction probability calculated here focuses on local interface confidence. This approach mitigates the limitations of ipTM, which can be substantially reduced by the presence of disordered regions or accessory domains that do not contribute to the core interaction interface (Dunbrack Jr. 2025).

After identifying potential candidate interacting partners for T3SS chaperones using AlphaFold, I modeled the complex structures using AlphaFold3 under six different configurations: each candidate was paired with either a single chaperone or a chaperone dimer, using one of three sequence segments—(i) the N‐terminal 100 amino acids, (ii) residues 10–140, or (iii) the full‐length protein. For example, a protein longer than 200 amino acids was modeled in six ways: full‐length paired with one or two chaperones, N‐terminal 100 residues paired with one or two chaperones, and residues 10–140 paired with one or two chaperones. I used 20 random seeds for each modeling configuration and generated 5 models for each random seed.

To identify common interaction patterns shared among experimentally characterized T3SS chaperone–effector and T3SS chaperone–regulator complexes, I used iAlign (Gao and Skolnick 2010) with command (ialign.pl. [pdb1] [pdb2] > [output]), a tool that calculates interface similarity independently of the global protein fold–an essential feature given the divergence of effectors and regulators. To determine whether the modeled chaperone–candidate interactions involved the N‐terminal β‐strand of the chaperone, I first aligned all chaperones used in structure modeling with FoldMansion (Gilchrist et al. 2026) to identify residues belonging to their N‐terminal β‐strands. Interacting residue pairs were defined as residues with any atoms within 5 Å distance between the chaperone and the candidate. If at least three residues from the N‐terminal β‐strand were among these interacting residues, the interaction was classified as involving this β‐strand.

To assess the presence of these candidates in *P. paraeruginosa*—a recently proposed nomenclature for a group of T3SS‐negative *P. aeruginosa* strains formerly classified under *P. aeruginosa*—I downloaded genome assemblies for *P. paraeruginosa* with an N50 above 50 Kb and screened them for T3SS using the TXSScan (Abby et al. 2016) T3SS model with command: “macsyfinder ‐‐db‐type ordered_replicon ‐‐sequence‐db [input_fn] ‐‐models‐dir TXSScan ‐‐models TXSScan bacteria/diderm/T3SS.” Among 71 genomes without T3SS, I clustered all annotated proteins using MMseqs2 with ‐c 0.8 (coverage >80%) and ‐‐min‐seq‐id 0.8 (sequence identity >80%). I identified reciprocal best hits for the final selected candidates between *P. aeruginosa* and *P. paraeruginosa* using BLASTP (Altschul 1997) and counted the ratio of cluster members in both species, respectively.

## CONFLICT OF INTEREST STATEMENT

The author declares no conflicts of interest.

## Supporting information


**Table S1.** Co‐existence of known chaprons and effectors/regulators.
**Table S2.** Final interacting candidates for T3SS chaperons homologs.

## Data Availability

The intermediate results and processing codes are available in http://prodata.swmed.edu/download/pub/T3SS_Pseudomonas_aeruginosa/intermediate_results.
